# REALizing and improving management of stable COPD in China: a multi-center, prospective, observational study to realize the current situation of COPD patients in China (REAL) – rationale, study design, and protocol

**DOI:** 10.1186/s12890-019-1000-x

**Published:** 2020-01-13

**Authors:** Ting Yang, Baiqiang Cai, Bin Cao, Jian Kang, Fuqiang Wen, Wanzhen Yao, Jinping Zheng, Xia Ling, Hongyan Shang, Chen Wang

**Affiliations:** 1 Department of Pulmonary and Critical Care Medicine, National Clinical Research Center for Respiratory Diseases, Institute of Respiratory Medicine, Chinese Academy of Medical Science, China-Japan Friendship Hospital, No 2, East Yinghua Road, Chaoyang District, Beijing, 100029 China; 2 Department of Respiratory Medicine, Peking Union Medical College Hospital, Beijing, China; 3 Department of Respiratory Medicine, The First Hospital of China Medical University, Shenyang, China; 4 Department of Respiratory Medicine, West China Hospital, Sichuan University, Chengdu, China; 5 Department of Respiratory Medicine, Peking University Third Hospital, Beijing, China; 6 Department of Respiratory Medicine, Guangzhou Institute of Respiratory Disease, 1st Affiliated Hospital of Guangzhou Medical College, Guangzhou, China; 7 Department of Medical Affairs, AstraZeneca China, Shanghai, China

**Keywords:** China, Chronic obstructive pulmonary disease, GOLD, Observational trial, Prospective trial, Real-world

## Abstract

**Background:**

Chronic obstructive pulmonary disease (COPD) is the fifth leading cause of death in China with a reported prevalence of 8.2% people aged ≥40 years. It is recommended that Chinese physicians follow Global Initiative for Chronic Obstructive Lung Disease (GOLD) and national guidelines, yet many patients with COPD in China remain undiagnosed. Furthermore, missed diagnoses and a lack of standardized diagnosis and treatment remain significant problems. The situation is further complicated by a lack of large-scale, long-term, prospective studies of real-world outcomes, including exacerbation rates, disease severity, efficacy of treatment, and compliance of COPD patients in China.

**Methods/design:**

The REALizing and improving management of stable COPD in China (REAL) study is a 52-week multi-center, prospective, observational trial. REAL aims to recruit approximately 5000 outpatients aged ≥40 years with a clinical diagnosis of COPD per GOLD 2016. Outpatients will be consecutively recruited from approximately 50 tertiary and secondary hospitals randomly selected across six geographic regions to provide a representative population. Patients will receive conventional medical care as determined by their treating physicians.

The primary objective is to evaluate COPD patient outcomes including lung function, health status, exacerbations, hospitalization rate, and dyspnea following 1 year of current clinical practice. Secondary objectives are to assess disease severity, treatment patterns, adherence to medication, and associated risk factors. Data will be collected at two study visits, at patients’ usual care visits, and by telephone interview every 3 months.

**Discussion:**

Knowledge of COPD among physicians in China is poor. The REAL study will provide reliable information on COPD management, outcomes, and risk factors that may help improve the standard of care in China. Patient recruitment began on 30 June 2017 and the estimated primary completion date is 30 July 2019.

**Trial registration:**

ClinicalTrials.gov identifier: NCT03131362. Registered on 20 March 2017.

## Background

Chronic obstructive pulmonary disease (COPD) is characterized by persistent respiratory symptoms and airflow limitation that is due to airway and/or alveolar abnormalities usually caused by significant exposure to noxious particles or gases [1]. Despite being a common, preventable, and treatable disease [1], COPD is the fifth leading cause of death in China [2]. The prevalence of COPD varies among geographic regions of China between 5 and 13% [3], with a reported prevalence of 8.2% among people aged ≥40 years [4].

The Chinese Thoracic Society recommends [5] that physicians follow Global Initiative for Chronic Obstructive Lung Disease (GOLD) [6] and national guidelines [5], yet adherence to guidelines among Chinese physicians is low [7]. Knowledge of COPD among Chinese physicians, including respiratory specialists, can be poor [8]. For instance, almost a quarter (22.1%) of respiratory physicians surveyed did not consider smoking to be the most significant risk factor for the development of COPD [8]. Furthermore, despite several large studies conducted over the last 10 years implicating biomass fuel as an important risk factor among Chinese patients with COPD [4, 9, 10], only 20.8% of respiratory physicians surveyed believe biomass fuel plays a critical role [8].

An observational study of respiratory diseases in the Asia-Pacific region included 210 patients with COPD from Taiwan but did not include patients from mainland China [11, 12]. Of these Taiwanese patients, the mean age was 71 and most (85.2%) were retired, there was a high proportion (78.6%) of “ever smokers”, but only 18.8% were current smokers [12]. Medication use was high (91.9%) in the preceding 4 weeks with mucolytics (44.8%) and methylxanthines (43.3%), the most frequently prescribed therapy, and direct medical costs were high (82.8% of direct costs) [12]. A recent study revealed that just over a quarter (26%) of newly diagnosed COPD patients surveyed in four major Chinese cities did not use any pharmacological treatment [13].

Many patients with COPD remain undiagnosed in China [4]. Raising awareness of COPD is the first step to improving rates of diagnosis, yet large-scale studies of real-world outcomes, exacerbation rates, and disease severity of COPD patients in China are lacking. Furthermore, there is a paucity of data derived from large Chinese cohorts regarding COPD maintenance and exacerbation treatment patterns, non-pharmacological management and risk factors for exacerbations, disease severity, and adherence.

The REALizing and improving management of stable COPD in China (REAL) trial aims to provide reliable information on COPD management, outcomes, and risk factors in China. Here we describe the rationale and design of this 52-week multi-center, prospective, observational study (NCT03131362, registered on 20 March 2017; protocol version 1.0, 9 July 2016). The primary objective is to evaluate COPD patient outcomes (including exacerbations) following 1 year of current clinical practice. Secondary objectives are to assess disease severity, treatment patterns, adherence to medication, and investigate associated risk factors. Clinical assessments will include spirometry, bronchial dilation and induced sputum tests, fractional exhaled nitric oxide (FENO), and chest computed tomography (CT) imaging. Patient reported outcomes will include the COPD Assessment Test (CAT) [14], modified Medical Research Council (mMRC) questionnaire [15] and COPD knowledge questionnaire (COPD-Q) [16]. Data on medication type, adherence, non-drug therapies, and total COPD treatment costs will also be collected.

## Methods/design

### Site selection

**Fig. 1:**
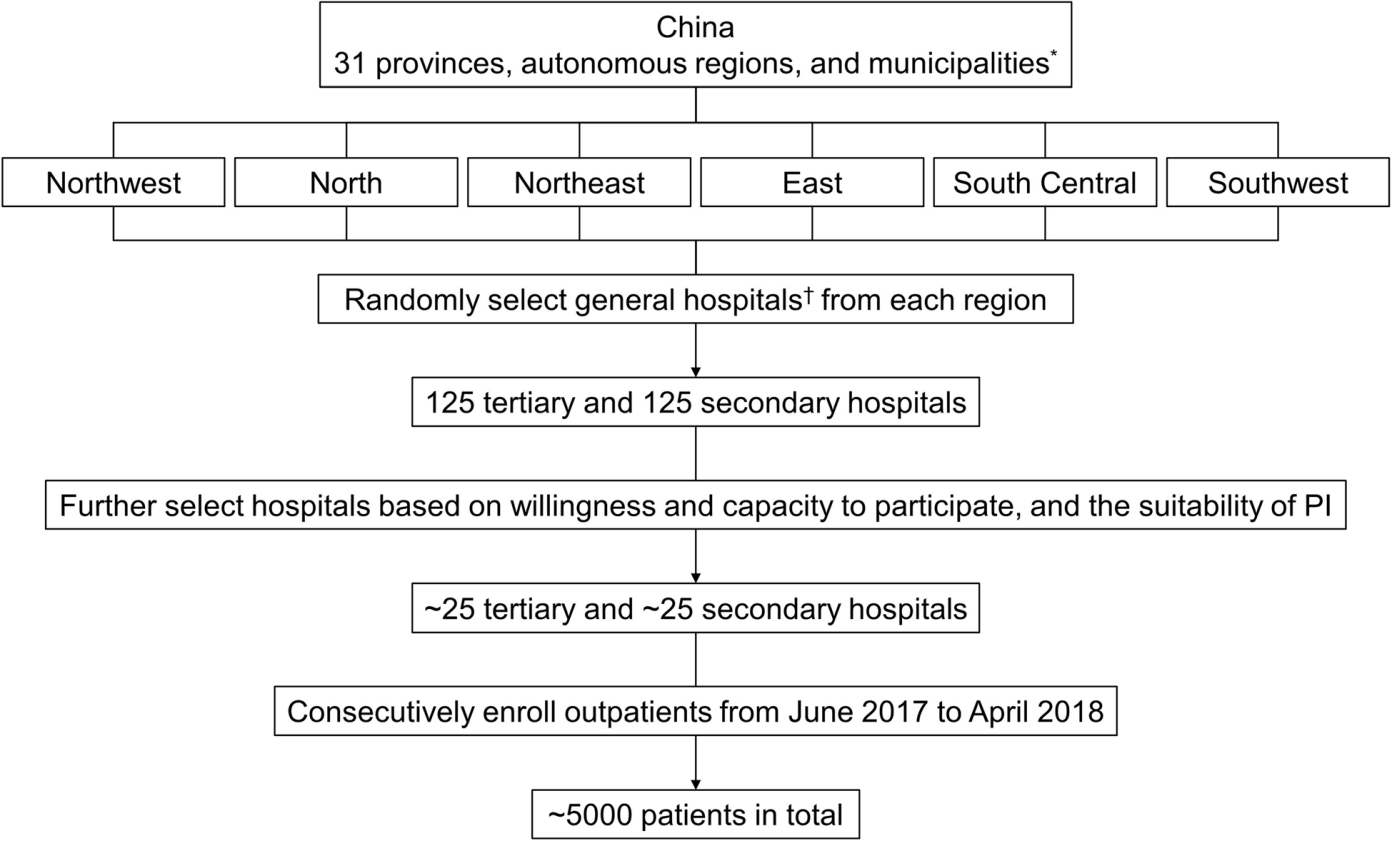
Multi-stage, stratified, cluster sampling method. * All secondary and tertiary hospitals administered by NHFPC. ^†^ Excluding traditional Chinese medicine hospitals; the actual hospital number and the ratio of tertiary to secondary hospitals in each region may be adjusted based on the hospital distribution in the region; subject numbers may be adjusted according to COPD outpatient numbers at the site

Patient recruitment began in June 2017 and the estimated primary completion date is 30 April 2019. To provide a representative population of Chinese patients with COPD, REAL aims to recruit approximately 5000 patients from approximately 50 tertiary and secondary hospitals across six geographic regions (north, northeast, east, south central, southwest, and northwest). A multi-stage, stratified, and cluster sampling method will be used to select a nationally representative sample of hospitals with respiratory departments (Fig. 1). Initially, 125 tertiary and 125 secondary hospitals administered by the National Health and Family Planning Commission (NHFPC) will be randomly selected from the six geographic regions. From this list, 25 tertiary and 25 secondary hospitals will be further selected based on their willingness and capacity to participate, and the suitability of the primary investigator. If the first round of sampling fails to yield enough sites, alternative hospitals will be selected by random selection of other sites in cities where hospitals declined to participate or further random sampling of the region.

### Patients

Eligible patients will be consecutively screened and enrolled in the study when they routinely visit their physician at a participating site between June 2017 and April 2018. Patients will be eligible for inclusion if they are outpatients aged ≥40 years, with a clinical diagnosis of COPD per GOLD 2016 [17]. The study will be performed in accordance with the Declaration of Helsinki and good clinical practice; as such, signed and dated informed consent will be obtained from every patient prior to participating in the study (see Additional file 1: Subject information and consent form). Exclusion criteria include participation in any interventional studies in the 30 days prior to enrollment and an acute exacerbation of COPD in the previous 4 weeks.

### Data collection

**Fig. 2:**
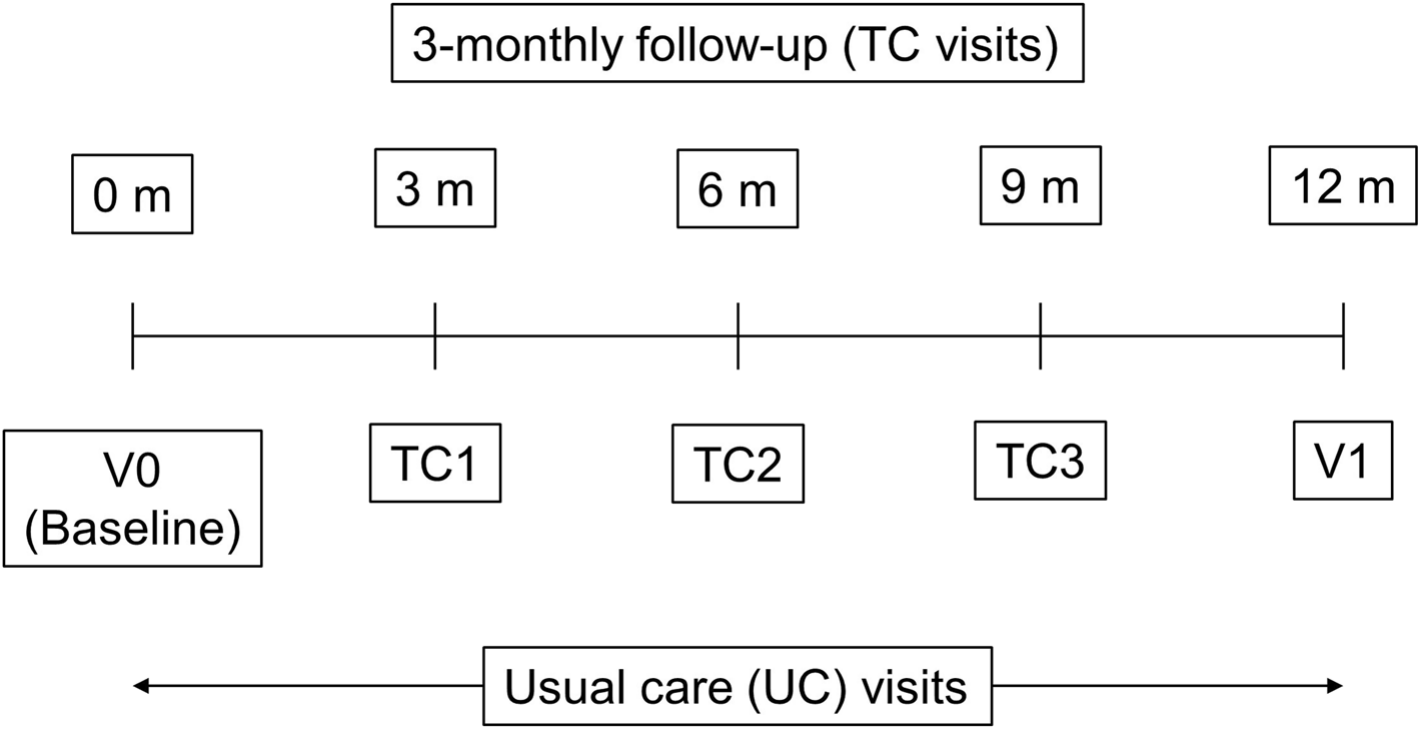
Study flow chart

There will be two prespecified study-specific visits at baseline (V0) and 12 months thereafter (V1). Telephone contact (TC) follow-ups will be arranged every 3 months following V0 (three TC follow-ups in total), during which investigators will call participants and gather study information (Fig. 2). Demographic data and baseline characteristics will be collected using case report forms (CRF) at V0. At V0, V1, and all TC follow-ups, symptoms, drug treatment, non-drug treatment, exacerbations, other respiratory diseases, comorbidities, complications, and direct cost will be collected from CRF and patient-reported outcome (PRO) questionnaires (mMRC and CAT) will be completed by the patient with minimal input from the investigator before any other procedures (questionnaires and stamped addressed envelopes will be provided in advance of TC follow-ups and investigators will remind participants to complete and return them during the call). If available at study visits, spirometry, chest CT imaging, induced sputum, and lab test data will be collected. With the exception of PRO, all the above study data will also be collected during patient’s usual care (UC) visits if available. Table 1 presents the Standard Protocol Items: Recommendations for Interventional Trials (SPIRIT) [18] figure for the REAL study (see also Additional file 2: SPIRIT checklist).

**Table 1:** Standard Protocol Items: Recommendations for Interventional Trials (SPIRIT) figure for the REAL study

Month	Enrollment	Data collection				
	0	3 ± 1	6 ± 1	9 ± 1	/	12 ± 1
Visit	V0	TC1	TC2	TC3	UC	V1
Informed consent	X					
Inclusion/exclusion criteria	X					
Demography (date of birth, ethnicity, gender, education, marital status, socioeconomic status, insurance status, residence area, occupation)	X					
Physical examination	X					X
Smoking history	X					
COPD history	X					
Disease severity GOLD 2016	X					X
Comorbidities, other respiratory diseases	X	X	X	X	X	X
Exacerbations	X	X	X	X	X	X
Symptoms	X	X	X	X	X	X
Spirometry and bronchial dilation if available	X				X	X
CAT, mMRC	X	X	X	X		X
COPD-Q	X					X
FENO if available	X				X	X
Chest CT imaging if available	X				X	X
Induced sputum test if available	X				X	X
Lab tests (blood gases, C-reactive protein and hematology) if available	X				X	X
COPD medications	X	X	X	X	X	X
COPD non-pharmacological treatment	X	X	X	X	X	X
Total COPD treatment expenses	X	X	X	X	X	X

### Endpoints

Disease severity will be measured by airway limitation (post-bronchodilator forced expiratory volume in 1 s, FEV_1_) and assessment of symptoms/risk of exacerbations (A/B/C/D) [17]. Exacerbations will be classified as mild: requiring an increase in rescue medication ≥3 puffs/day for ≥2 consecutive days; moderate: requiring systemic glucocorticosteroids and/or antibiotics; or severe: hospitalization, emergency room visit, or leading to death [19]. Endpoints are given in Table 2.

**Table 2:** Endpoints of REAL

Endpoints	Outcome measures
Primary	Mean rate of acute exacerbations (per patient per year)^a^ Proportion of patients hospitalized due to exacerbation^a^ Distribution of COPD exacerbations by severity Mean reduction from baseline of available FEV_1_^a^ Mean change in CAT total score Mean change in mMRC score Mean change in COPD-Q total score
Secondary	Distribution of disease severity at baseline and V1 Distribution of COPD medication by drug class^b^ at baseline and V1 Distribution of maintenance medication^a^ and dosage by disease severity at baseline and V1 Distribution of maintenance medication^b^ at each usual visit Distribution of medications for exacerbations Distribution of non-drug treatments (health education, smoking cessation, physical activity, and vaccination) Medication compliance Visit compliance Mean total direct cost of COPD management over 1 year
Exploratory	Risk factors for exacerbations Risk factors for disease severity Risk factors for medication compliance

^a^ Also stratified by disease severity at baseline

^b^*ICS* Inhaled corticosteroid, *LABA* long-acting β_2_-agonist, *ICS/LABA* combined long-acting β_2_-agonists plus corticosteroid, *SABA* short-acting β_2_-agonist, *SAMA* short-acting muscarinic antagonist, *SABA/SAMA* combined short-acting β_2_-agonist plus short-acting muscarinic antagonist, *LAMA* long-acting muscarinic antagonist, methylxanthines, mucolytics, traditional Chinese medicine, and others (antibiotics, systemic corticosteroids, vaccines, antioxidant agents, etc.)

### Sample size estimate

Assuming the exacerbation rate is 1.5 per person per year, 3500 subjects will enable a predefined limit of precision of 0.041 (half the width of 95% confidence interval [CI]). Based on a 30% patient drop-out rate, overall 5000 subjects will be required.

### Safety assessments

As a non-interventional study with no specific AstraZeneca drug under investigation, there will be no proactive safety data collection. Any adverse events considered related to any AstraZeneca products will be reported to health authorities as per local regulations and to the AstraZeneca China patient safety group.

### Statistical analysis

As an observational study, there will be no pre-planned hypothesis testing, and data analysis will be primarily descriptive in nature. Summary statistics will be presented for both primary and secondary endpoints. For continuous variables, these will include the number, mean, median, standard deviation (SD), minimum values, and maximum values. For categorical variables, these will include frequency counts and percentages for each category. The rate of exacerbations per year will be estimated under the assumption of Poisson distribution. The mean and standard error of the overall exacerbation rate per year will be presented. To reduce any potential regional bias, stratified and weighted summaries will be calculated using the proportions of the regional and total populations if applicable. A similar approach may also be applied with respect to sites. COPD treatments will be summarized by drug class and by treatment summaries may be performed. Risk factors for exacerbations, disease severity, and medication compliance will be analyzed using Poisson regression and multinomial regression and presented as odds ratios and 95% CI.

## Discussion

In China, many clinicians and patients have a low level of knowledge of the management, prevention, and risk factors for COPD [7, 20, 21]. This represents a growing problem as COPD mortality and disease burden are predicted to increase worldwide over the coming years [22]. The high occurrence of COPD in China may be due to greater exposure to risk factors, such as smoking [23] and biomass fuel [9]; however, other factors may be revealed to be important by the REAL study.

Despite the endorsement of GOLD recommendations by the Chinese Thoracic Society [5], there have been concerns raised as to whether they are appropriate or feasible for use in Chinese clinics [24]. With respect to diagnosis and classification of disease severity, one problem is the lack or incorrect use of spirometry testing [24]. Studies have shown that in tertiary hospitals only 50% of patients with COPD have undergone spirometry testing, in primary and secondary hospitals the proportion was as low as 18% [25], and in some rural areas spirometry testing is not conducted at all [26]. Nevertheless, when spirometry testing is carried out, the prognostic validity of GOLD 2011 [6] criteria – that differ only slightly from GOLD 2016 criteria which also take into account hospitalization for exacerbations – has been demonstrated in Asian patients with COPD [27].

Dyspnea is characteristic of COPD but it is now accepted as only one of several symptoms that affect patients, and comprehensive assessments are required to give a full picture of disease burden [28]. As commentators have noted, high patient volumes and staff shortages in Chinese clinical practice mean that simple methods of assessment are preferred and often judged to be the best [24]. The REAL study has opted to use the mMRC [15] and the CAT [14] to assess the level of health status impairment. The mMRC provides a simple yet reliable way to categorize the levels of disability due to breathlessness experienced by patients with COPD [29]. The CAT is an 8-item measure of health status impairment in COPD, in which questions are scored from 0 to 5 [14]. The CAT has been used in many trials [30], it shows good agreement with the more complex and time consuming St George’s Respiratory Questionnaire (SGRQ) [31, 32], and in a large study of Chinese patients it correlated moderately with the mMRC dyspnea score [33]. The COPD-Q [16] is a reliable tool that can be used to test disease knowledge among with patients with limited health literacy [34]. As the questionnaire is composed of only 13 simple questions with “true or false” answers, the COPD-Q can be easily translated into other languages [35].

The majority of recent studies of health-related costs among Chinese patients with COPD have focused on specific interventions such as mechanical intervention [36], vaccination [37], or specific risk factor such as pollutants [38, 39]. One study conducted in 2011 in four Chinese cities among 678 patients with COPD found that poorer quality of life as determined by the EuroQol-5 dimensions (EQ-5D) health questionnaire was significantly linked to increased medical costs [40]. A further analysis of this cohort showed the mean annual direct medical (DMC), direct non-medical (DNMC), and indirect (IC) costs per COPD patient were 11,968 yuan (US$ 1853), 539 yuan (US$ 83), and 2087 yuan (US$ 323), respectively, with over half (56.7%) of total costs due to hospitalization [41]. While these data are somewhat dated, they do offer some degree of insight into the high cost burden of COPD in China and will be interesting to compare with the results from the larger REAL cohort.

The Adelphi respiratory disease-specific program (DSP) utilized surveys conducted in 2010 in China (*N* = 511) and 2013 in Europe (*N* = 1242) and the United States (*N* = 413) to provide information on pharmacological management of COPD [42]. In the Chinese sample, the most prescribed maintenance treatment was inhaled corticosteroid/long-acting β_2_-agonist (ICS/LABA) dual therapy (25.9%), followed by ICS/LABA plus xanthines (20.2%), other (unspecified) therapies (16.5%), xanthines only (11.2%), and ICS/LABA plus leukotriene receptor antagonist (LTRA) (10.6%) [42]. The level of ICS/LABA use was comparable to that of the American cohort (25.9%) and higher than that of the European cohort (15.7%); however, less than 1% of the American and European patients used ICS/LABA plus xanthines, xanthines only, or ICS/LABA plus LTRA, with 4.4 and 5.6% using ‘other’ treatments, respectively [42]. The high use of ‘other’ therapies among the Chinese patients may, in part, be attributable to the high rate of non-prescription medicine use that has been observed [43]. The most common maintenance therapy among American and European patients was triple therapy (long-acting muscarinic antagonist [LAMA] plus ICS/LABA or LAMA plus ICS plus LABA) in 33.2 and 33.1% of patients, respectively, but only 1.6% of Chinese patients reported triple therapy use [42]. Triple therapy is recommended by GOLD for group D patients (high symptom burden and high exacerbation risk) [1]. The Adelphi respiratory DSP survey in China was carried out in 2010 [42], before the endorsement of the GOLD guidelines by the Chinese Thoracic Society in 2013 [5]. The REAL study will be the first major investigation of treatment patterns since the introduction of the new guidelines; however, as adherence to guidelines among Chinese physicians is low [6], it remains to be seen what level of impact this will have.

One strength of the REAL study is the 52-week duration and large sample size which, to our knowledge, is unprecedented among prospective observational studies of COPD conducted in mainland China to date. A steering committee of respiratory experts (TY, BCai, BCao, JK, FW, JZ, XL, and CW) was consulted on protocol design and data collection, and will oversee operational aspects of the study. Multi-stage random sampling and selection of sites will ensure study participants are representative of the six regions. Over half of exacerbations may be unreported [44], so the use of investigator-led telephone follow-ups during the REAL study may prompt participants to provide a more complete picture of the pattern of their exacerbations. Potential limitations include the fact that since patients will be enrolled only from secondary and tertiary hospitals, the sample may not be representative of Chinese COPD patients as a whole. Patients from secondary and tertiary hospitals are less likely to be lost to follow-up over the course of the trial than patients in primary care. However, as with any observational study, withdrawal of patients and missing data may introduce bias and there may be further unidentified or unmeasured confounding variables.

In conclusion, the REAL study should help to provide reliable information on COPD management, outcomes, and risk factors that may help improve the standard of care in China.

### Trial status

ClinicalTrials.gov identifier: NCT03131362. Registered on 20 March 2017; protocol version 1.0, 9 July 2016. The first patients were recruited to REAL on 30 June 2017 and the estimated primary completion date is 31 January 2020.

## Supplementary information

**Additional file 1.** Subject information and consent form.

**Additional file 2.** SPIRIT checklist.

## Data Availability

The datasets generated and/or analyzed during the current study will be available via the AstraZeneca Group of Companies – Data Request Portal at: https://astrazenecagroup-dt.pharmacm.com/DT/Home More information on AstraZeneca’s clinical trials disclosure policy is available at: http://astrazenecagrouptrials.pharmacm.com//ST/Submission/Disclosure. This report adheres to the SPIRIT guidelines. A completed SPIRIT checklist is available in Additional file 2.

## Abbreviations

CAT: COPD Assessment Test
COPD: Chronic obstructive pulmonary disease
COPD-Q: COPD knowledge questionnaire
CRF: Case report form
CT: Computed tomography
DMC: Direct medical cost
DNMC: Direct non-medical cost
DSP: Disease-specific program
EQ-5D: EuroQol-5 dimensions
FENO: Fractional exhaled nitric oxide
FEV_1_: Forced expiratory volume in 1 s
GOLD: Global Initiative for Chronic Obstructive Lung Disease
IC: Indirect cost
ICF: Informed consent form
ICS: Inhaled corticosteroid
LABA: Long-acting β_2_-agonist
LAMA: Long-acting muscarinic antagonist
LTRA: Leukotriene receptor antagonist
mMRC: Modified Medical Research Council
NHFPC: National Health and Family Planning Commission
PRO: Patient-reported outcome
SABA: Short-acting β_2_-agonist
SAMA: Short-acting muscarinic antagonist
SGRQ: St George’s Respiratory Questionnaire
TC: Telephone contact
UC: Usual care
V0: Baseline visit
V1: Visit at 12 months
