# Supplementary material for: REALizing and improving management of stable COPD in China: a multi-center, prospective, observational study to realize the current situation of COPD patients in China (REAL) – rationale, study design, and protocol
Source: BMC Pulm Med. 2020 Jan 13;20:11. doi: 10.1186/s12890-019-1000-x (PMC6958695; doi:10.1186/s12890-019-1000-x)
Supplement: Supplementary file 1 — Additional file 1. Subject information and consent form. [file 12890_2019_1000_MOESM1_ESM.pdf]

OBSERVATIONAL STUDY PRIMARY SUBJECT INFORMATION AND CONSENT FORM

Observational Study Name/Code: D2287R00114 Subject Initials:
Site No: Screen Code:

WHY HAVE I BEEN GIVEN THIS FORM?

You are being asked to take part in the REALizing and improving management of stable Chronic Obstructive Pulmonary Disease (COPD) in China——a multi-centre, prospective, observational study to realize the current situation of COPD patients in China (REAL I). Before you decide if you want to take part, it is important for you to understand why this study is being done, how your information will be used, what the study will involve and the possible benefits, risks and discomforts. Please take the time to read the following information carefully and discuss it with your family member and study doctor or study nurse. Please indicate if you are already participating in any other study. In this case, to decide whether you can take part in the REAL I study, please discuss the study you are currently participating in with your doctor. This Informed Consent Form (ICF) is in duplicate. If you participate, you will receive one ICF.

WHAT IS THE BACKGROUND AND PURPOSE OF THE STUDY?

Chronic obstructive pulmonary disease (COPD) prevalence in China has been reported as 8.2% in general population aged 40 and older.3This figure is likely to be an underestimate as many patients with COPD remain undiagnosed. COPD is still the fourth leading cause of death in China. COPD mortality and burden are predicted to increase in the coming decades. Its huge social and economic impact makes COPD a major health problem in China. This multi-centre, prospective, observational study can help to understand the current COPD situation and may provide some references for improving COPD evaluation and treatment strategies in China.

The objective of this study is to describe the one year clinical outcome of COPD patients in current clinical practice in China; To describe the severity distribution of COPD patients in China; To describe the treatment pattern among COPD patients in China; To assess the treatment compliance of COPD patients in China. To investigate the risk factors of COPD exacerbations, severity and compliance of COPD patients.

DO I HAVE TO TAKE PART?

It is up to you to decide whether or not to take part. Even if you decide not to participate in the study, you will not be disadvantaged in any way, including medical treatment and care you are entitled to receive. If you do decide to take part you will be given this Informed Consent Form to sign. If you decide to take part, you are still free to withdraw at any time. This will not affect the standard of care you receive. If you withdraw, you will be asked about the reason for withdrawal and other relevant study data if you agree to answer. No further study related contacts or data collection will then occur.

The study sponsor may stop the study for administrative reasons unrelated to the purpose of this study. In such a case, you will be informed immediately.

WHAT DO I HAVE TO DO?

This is a multi-centre, prospective, observational study carried out in China, covering 50 sites and 5000 patients totally.

During your routine clinical practice in respiratory outpatient department, the Investigator will firstly explain the purpose of the study to you and written informed consents will be obtained. If you decide to participate in this study, the following procedures will be performed:

- Determination of eligibility by checking the inclusion/exclusion criteria
- If you are qualified for this study, there will be 2 times on-site visits and 3 times telephone call visits in total. 2 on-site visit including: the screening/baseline visit (the 1st time on-site visit) and 12 months later/study end (the 2nd time on-site visit). 3 times telephone call visits including: the telephone call visits 3 months after enrolment, the telephone call visits 6 months after enrolment, the telephone call visits 9 months after enrolment. Following the clinical practice, the investigator will ask you to come back to the sites for usual care purpose, data will also be collected at these usual care visits, where applicable, with no pre-specified visit number.

- The following information will be collected according to the study protocol:
  - Demographic variable (Birthday, Ethnicity, Marriage status, Educational level, etc.);
  - Physiological variables (Height and weight, Waist circumference, Heart rate;
  - COPD information (Disease severity, Age at first diagnosis of disease including chronic bronchitis, emphysema or COPD, Personal and family history of COPD, Comorbidities, Occurrences and timing of COPD complications, Exacerbations including the previous 12 months before enrolment, Occurrences and timing of other respiratory infections and respiratory diseases, Symptoms: coughing, sputum production, breathlessness, chest tightness and wheezing, Patient Reported Outcome(PROs): COPD Assessment Test(CAT), Modified British Medical Research Council (mMRC), and COPD knowledge Questionnaire (COPD-Q), Lung function measurements including spirometry and bronchodilator reversibility test (within 6 months before visits will be collected if available), Fractional Exhaled Nitric Oxide (FENO), Chest Computed Tomography (CT) imaging,

Induced sputum test, Lab tests such as blood gas analysis, C-reactive protein and haematology, if available. etc.);

- COPD treatment status (Drug-treatment, None drug-treatment -health education, smoking cession, respiratory function exercise and vaccine injection and total cost, etc.)

WHAT ARE THE POSSIBLE RISKS AND BENEFITS OF TAKING PART?

This study will only capture routine medical practice as provided to you by your health care provider. Your therapeutic strategy is not decided in advance by this study but falls within current practice. Therefore, there is no specific risk related to your participation in the study.

Participating in this study, there is no immediate clinical benefit for you, but you can have a deeper understanding of your conditions and treatments. The information we get from this study may help us to describe how COPD patients are managed in real-life practice, and to increase our knowledge about the disease and its symptoms. This will hopefully help us to better treat patients with COPD in the future.

DO I HAVE TO PAY OR WILL BE PAID TO TAKE PART IN THE STUDY?

You will not pay or receive payment for participating in this study.

WILL INFORMATION ABOUT ME BE KEPT CONFIDENTIAL?

By signing this form you consent to the Study Doctor and his or her staff collecting and using personal data about you for the study ('Study Data'). This includes: your date of birth, your sex, your ethnic origin and personal data on your physical or mental health or condition. Your consent to the use of Study Data does not have a specific expiration date, but you may withdraw your consent at any time by notifying the Study Doctor.

The Study Data shared with the Sponsoring Company is protected by the use of a code which is a number specific to you. The Study Doctor is in control of the Code key, which is needed to connect Study Data to you. A person appointed by the Sponsoring Company, regulatory authorities or other supervisory bodies may review any Study Data held by the Study Doctor.

The Study Doctor will use Study Data to conduct the Study. The Sponsoring Company may use Study Data to conduct the Study, to support applications for approval of targeted or other study medication, for research related to the development of targeted or other pharmaceutical products, diagnostics or medical aids, and for further research on the targeted or other diseases. The Study Doctor's institution and the Sponsoring Company are each responsible for their handling of Study Data in accordance with applicable Data Protection law(s).

The Sponsoring Company may share Study Data with other companies within its group, with its service providers, its contractors and with research institutions, and research based commercial organisations who will use Study Data only for the purposes described above. Also, the Study Data from this study may be analyzed together with other parallel studies to be conducted in other countries across the world.

When your Study Data is processed in Sweden by the Sponsoring Company, AstraZeneca AB is responsible for your personal data.

The Sponsoring Company may transfer Study Data to countries outside of China for the purposes described in this document. Please be aware that the laws in such countries may not provide the same level of data protection as in China and may not stop Study Data from being shared with others. All that is transferred will be coded. Please note, the results of the study may be published in medical literature, but you will not be identified.

You have the right to request information about Study Data held by the Study Doctor and Sponsoring Company. You also have the right to request that any inaccuracies in such data be corrected. If you wish to make a request, then please contact the Study Doctor, who can help you contact the Sponsoring Company if necessary.

If you withdraw your consent the Study Doctor will no longer use Study Data or share it with others. The Sponsoring Company may still use Study Data that was shared with it before you withdrew your consent.

By signing this form you consent to the use of Study Data as described in this form.

WHAT IF I HAVE QUESTIONS?

Should you need additional information, please contact:

Name: Telephone(+86):

Address :

If you have questions about your rights as a research participant, you should contact the individual below.

Name: Telephone: (+86)
Address:

INFORMED CONSENT STATEMENT

Subject agreed statement:

I have received verbal information on the above study and have read the attached written information.

I have been given the chance to discuss the study, ask questions and received answers for any questions.

I consent to take part in the study and I am aware my participation is entirely voluntary.

I understand that I may withdraw at any time without this affecting my future care.

By signing this information and consent form I agree that my personal data, including data relating to my physical health or condition, and race or ethnic origin, may be used as described in this consent form, including transfer to countries outside of China.

I understand I will receive a copy of this information and consent form.

________________________________ __________________________

Signature of subject Date of Signature

____

Printed name of subject (BLOCK CAPITALS)

Investigator agreed statement:

I confirm to give the subject detailed explanation about the content of this study, including subjects' possible risks and discomforts, and give subject a signed copy of this information and consent form.

________________________________ __________________________

Signature of person conducting the informed consent discussion Date of Signature

____

Printed name of person conducting the informed consent discussion (BLOCK CAPITALS)

The legally acceptable representative signature should be added if the subject is a minor, or is unable to sign for him or herself. The relationship between the subject and the legally acceptable representative should be stated.

______________________________	__________________________
Signature of legally accepted representative	Date of Signature

____

Printed name of legally accepted representative (BLOCK CAPITALS)

____

Relationship of legally accepted representative to subject (BLOCK CAPITALS)

The impartial witness signature should be added if the subject is unable to read or write.

______________________________	__________________________
Signature of witness	Date of Signature

____

Printed name of witness (BLOCK CAPITALS)
